# Advances in the molecular pathogenesis and cell therapy of stress urinary incontinence

**DOI:** 10.3389/fcell.2023.1090386

**Published:** 2023-02-08

**Authors:** Xiao-xiao Wang, Lei Zhang, Ye Lu

**Affiliations:** Department of Obstetrics and Gynecology, Peking University First Hospital, Beijing, China

**Keywords:** stress urinary incontinence, molecular pathogenesis, stem cell therapy, gene regulation, exosome differentiation

## Abstract

Stress urinary incontinence (SUI) is very common in women. It affects patients’ mental and physical health, and imposed huge socioeconomic pressure. The therapeutic effect of conservative treatment is limited, and depends heavily on patient persistence and compliance. Surgical treatment often brings procedure-related adverse complications and higher costs for patients. Therefore, it is necessary to better understand the potential molecular mechanisms underlying stress urinary incontinence and develop new treatment methods. Although some progress has been made in the basic research in recent years, the specific molecular pathogenic mechanisms of SUI are still unclear. Here, we reviewed the published studies on the molecular mechanisms associated with nerves, urethral muscles, periurethral connective tissue and hormones in the pathogenesis of SUI. In addition, we provide an update on the recent progresses in research on the use of cell therapy for treating SUI, including research on stem cells therapy, exosome differentiation and gene regulation.

## 1 Introduction

Stress urinary incontinence (SUI) is the most common type of urinary incontinence and is defined as the involuntary leakage of urine when bladder pressure exceeds urethral closure pressure due to physical exertion, exercise, coughing or sneezing (D'Ancona et al., 2019). The prevalence of SUI worldwide ranges widely from 10% to 70%, owing to different survey methodologies and study populations (Capobianco et al., 2018). In the United States, the prevalence of SUI in 2017–2020 was 46% among adult women and 31% among adult men with prior diagnoses of prostate and bladder cancer (Abufaraj et al., 2021; Cao et al., 2022). In China, the prevalence of SUI in women aged 20 years and above reached 18.9%, and SUI affected over one-third of the perimenopausal women (Li T. et al., 2019). The prevalence of SUI increases with age, and the peak incidence is in postmenopausal and pregnant women. Several other factors are related to SUI risk, including delivery, menopause, strenuous physical work, smoking, obesity and chronic cough (Tan et al., 2018).

Although SUI is not a life-threatening disease, it results in physical, social, and psychological adverse consequences, and leads to low self-esteem, impaired quality of life and social isolation for patients. Untreated SUI is also associated with falls and fractures, sleep disturbances, depression, and urinary tract infections (Lukacz et al., 2017). In addition, SUI contributes to a significant financial burden to the health system, with direct costs exceeding $12 billion in the United States alone (Chong et al., 2011). As the world’s population ages, the prevalence and management costs of SUI will increase in the next few decades.

There are two main mechanisms of SUI. The first is urethral hypermobility, in which the urethra and bladder neck fail to close adequately when abdominal pressure increases due to the loss of supportive structures of the pelvic floor, resulting in the leakage of urine. The second is intrinsic urethral sphincter deficiency, which results in poor urethral closure due to the loss of the urethral mucosa and muscle tone (Wu, 2021). However, the molecular pathogenic mechanisms in SUI are still unclear. In this review, we mainly summarized the recent progresses in research on the molecular mechanisms of SUI pathogenesis and the use of cell therapy in SUI treatment.

## 2 Neurogenic factors

**FIGURE 1:**
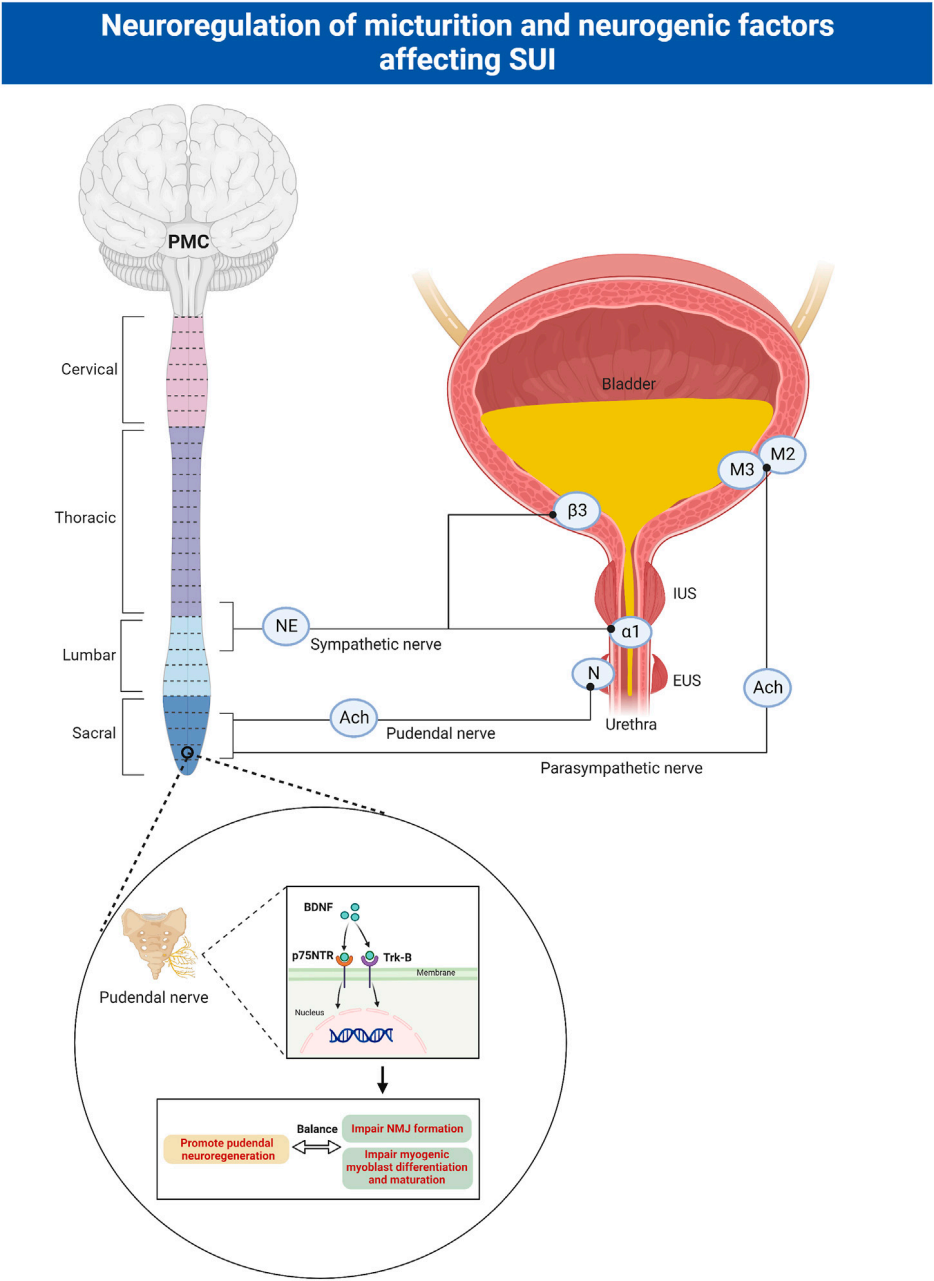
Neuroregulation of micturition and neurogenic factors affecting SUI. The parasympathetic nerve stimulates the bladder detrusor muscle, mediated by muscarinic receptors (M2, M3) being activated by ACh. The sympathetic nerve stimulates urethral smooth muscle and inhibits bladder detrusor, mediated by α1-adrenergic and β3-adrenergic receptors, respectively. The somatic pudendal nerve stimulates striated muscle of the external urethral sphincter, mediated by ACh activating N receptors. BDNF binds to Trk receptors and p75NTR receptors to facilitate regeneration of pudendal nerve, but impair NMJ formation and myogenic myoblast differentiation and maturation. ACh: acetylcholine; EUS: external urethral sphincter; IUS: internal urethral sphincter; SUI: stress urinary incontinence; PMC: pontine micturition center; BDNF: brain-derived neurotrophic factor; Trk: tropomyosin receptor kinase; p75NTR: pan neurotrophin receptors; NMJ: neuromuscular junctions.

The lower urinary tract, composed of the bladder and urethra, is responsible for the excretion and storage of urine. The parasympathetic nerve stimulates the bladder detrusor muscle, mediated by muscarinic receptors (M2, M3) being activated by acetylcholine (ACh). The sympathetic nerve stimulates urethral smooth muscle and inhibits bladder detrusor, mediated by α1-adrenergic and β3-adrenergic receptors, respectively. The somatic pudendal nerve stimulates striated muscle of the external urethral sphincter (EUS), mediated by ACh activating N receptors (Yoshimura and Miyazato, 2012). During bladder filling, the parasympathetic innervation of the detrusor is inhibited, and the urethral smooth and striated sphincter are activated. When urinating, multiple parts of the brain are activated and generate a series of signals that ultimately lead to the activation of the pontine micturition center, which activates the sacral parasympathetic nerves, leading to bladder contraction, urethra relaxation and urine outflow (Fowler, 2006) (Figure 1). If a nerve in any one of these three systems is damaged by trauma, childbirth, or iatrogenic causes, it results in symptoms of SUI (Balog et al., 2019).

SUI mainly occurs when the intravesical pressure exceeds the urethral pressure during stress moments. Passive as well as active urethral closure mechanisms are important for continence. Passive closure mechanisms can physiologically only occur on the proximal urethra, and the neurally mediated active closure mechanisms on the distal two-thirds of the urethra play an important role in the continence (de Vries et al., 2018). However, the active urethral closure mechanism is damaged in SUI patients as evidenced by a series of studies. These mechanisms include lower cough-induced leak point pressure (LPP), disappearance of the pressure increment in the urethra preceding cough, and a loss of voluntary increases in urethral pressure (Yoshimura et al., 2008). In addition, the urethral closure mechanism activated by a sudden increase in abdominal pressure is mediated by the somatic nerves and is under central nervous control *via* Onuf’s nucleus (Kamo et al., 2003). Normal people activate the pudendal nerve (PN) during sneezing, stimulate the contraction of urethral muscle and avoid of incontinence. While the active urethral continence reflex induced by sneezing may be impaired in women with SUI, due to the PN damage caused by childbirth (Kamo et al., 2006).

### 2.1 Neurotrophins

Neurotrophins (nerve growth factor (NGF), brain-derived neurotrophic factor (BDNF), neurotrophin-3 (NT-3), and neurotrophin-4 (NT-4)), mainly produced by Schwann cells, are widely recognized for their roles in promoting cell growth, survival and differentiation in several classes of neurons (Chang et al., 2019; Gordon, 2020). Neurotrophins bind to two main classes of receptors-tropomyosin receptor kinase (Trk) receptors and pan neurotrophin (p75NTR) receptors. The p75NTR receptors belongs to the tumor necrosis factor receptor family. They can bind to all neurotrophins and participate in apoptosis (Song et al., 2014). Individual neurotrophins bind specifically to Trk receptors, causing receptor dimerization and autophosphorylation of tyrosine residues. This lead to the activation of downstream signaling cascades including phosphatidylinositol 3-kinase (PI3K)/protein kinase B (Akt), Ras-mitogen-activated protein kinase (Ras-MAPK), and phospholipase Cγ (PLCγ). These pathways have known roles in neuronal survival, axonal outgrowth, and synaptic plasticity (Song et al., 2014).

Clinical studies have shown that serum BDNF levels are significantly reduced in the 30th to 37th weeks of pregnancy and remain low for 2–3 months postpartum (Lommatzsch et al., 2006; Vega et al., 2011). The specific mechanism leading to this phenomenon is still unclear. After peripheral nerve injury, BDNF is upregulated in innervated target organs and axons distal to injury sites (Omura et al., 2005), and the use of BDNF has shown promising results in improving functional and anatomic recovery (Simon et al., 2003). Some studies have shown that BDNF expression increases in the EUS 1 day after pudendal nerve crush (PNC) in rats, promoting pudendal neuroregeneration *via* the retrograde transport of BDNF from the EUS to its innervating motor neuron cell bodies within Onuf’s nucleus (Pan et al., 2009). However, BDNF levels in the EUS are decreased in an animal model of SUI that simulates childbirth injury. Despite facilitating the regeneration of PNs, BDNF is detrimental to muscular recovery and development since it impairs neuromuscular junction (NMJ) formation as well as myogenic myoblast differentiation and maturation (Mousavi and Jasmin, 2006). The concurrent injury of the EUS muscle and the PN during childbirth results in the downregulation of BDNF in the EUS. This enables sphincter muscle and NMJ repair but likely impairs the PN neuroregenerative response. Direct application of BDNF to the pudendal nerve accelerates continence recovery through enhancing pudendal nerve recovery and facilitating the recovery of EUS anatomy and function in vaginal distension (VD) and PNC models (Gill et al., 2013). Electrical stimulation has been shown to upregulate the expression of BDNF in motor neurons and facilitate axon regeneration through an increase in βII-tubulin expression after injury (Jiang et al., 2013).

### 2.2 Sympathetic neurotransmitters

The excitatory pathway responsible for maintaining smooth muscle contraction in the urethra and bladder neck originates from sympathetic preganglionic neurons (Anderson, 1993). Pharmacological experiments have shown that the bladder sympathetic reflex pathway is regulated by the central noradrenergic system (Danuser et al., 1995). Norepinephrine (NE) stimulates urethral and bladder neck smooth muscle contraction *via* α1-adrenoceptors, and causes detrusor relaxation *via* β2-adrenoceptors or, most predominantly, *via* β3-adrenoceptors (Nomiya and Yamaguchi, 2003). Sympathetic activity can be inhibited by α 1-adrenergic receptor blockers or α 2-adrenergic receptor (AR) agonists. In addition, serotonin and norepinephrine can enhance pudendal nerve activity through glutamate release in Onuf’s nucleus, resulting in increased EUS tone. Serotonin also regulates collagen synthesis by activating transforming growth factor-β1 (TGF-β1). TGF-β1 is a key cytokine in extracellular matrix production, cell proliferation and fibrosis (Song et al., 2016). These neurotransmitters are being investigated as potential targets for the treatment of SUI.

Duloxetine, a dual 5-hydroxytryptamine (HT) and norepinephrine reuptake inhibitor, can stimulate pudendal motor neurons and increase EUS and pelvic floor muscle contractility (Dhaliwal et al., 2022). Duloxetine can reduce the frequency of incontinence episodes in SUI compared to placebo (Norton et al., 2002; Millard et al., 2004; van Kerrebroeck et al., 2004), and is the first drug in the world to be approved for the treatment of women with moderate-to-severe SUI. A study showed that the α2- AR blocker imidazoxan could enhance the effect of duloxetine on the sneeze-induced urethral continence reflex in rats, and this combination therapy may be an effective new treatment for SUI (Norton et al., 2002). Studies on 5-HT receptors showed that intrathecal administration of 8-hydroxy-2-(di-n-propylamino)-tetralin, a 5HT-_1A_ agonist, reduced the urethral constriction response during sneezing by 48.9%, whereas when 5HT-_2B/2C_ was administered, the agonist meta-chlorophenylpiperazine (mCPP) increased this response (Miyazato et al., 2009). These findings suggest that 5HT-_2C_ activation of the receptor could actively enhance urethral closure during sneezing. In addition to duloxetine, imipramine, which is a norepinephrine and serotonin reuptake inhibitor, is thought to improve urethral smooth muscle contraction for the treatment of SUI or mixed urinary incontinence (Cipullo et al., 2014). Moreover, venlafaxine (0.1–10 mg/kg) and S-norfluoxetine (0.01–10 mg/kg) have also been shown to increase bladder volume, but they have not shown a clear therapeutic effect on SUI. Clenbuterol hydrochloride, a beta-AR agonist, increases periurethral striated muscle contraction by releasing acetylcholine at the NMJ and has been shown to be effective in the treatment of SUI (Alhasso et al., 2005). However, there have been few related studies, and this agonist has not been widely used. Drug development targeting 5-HT and adrenergic mechanisms requires further research.

## 3 Muscle-derived factors

**FIGURE 2:**
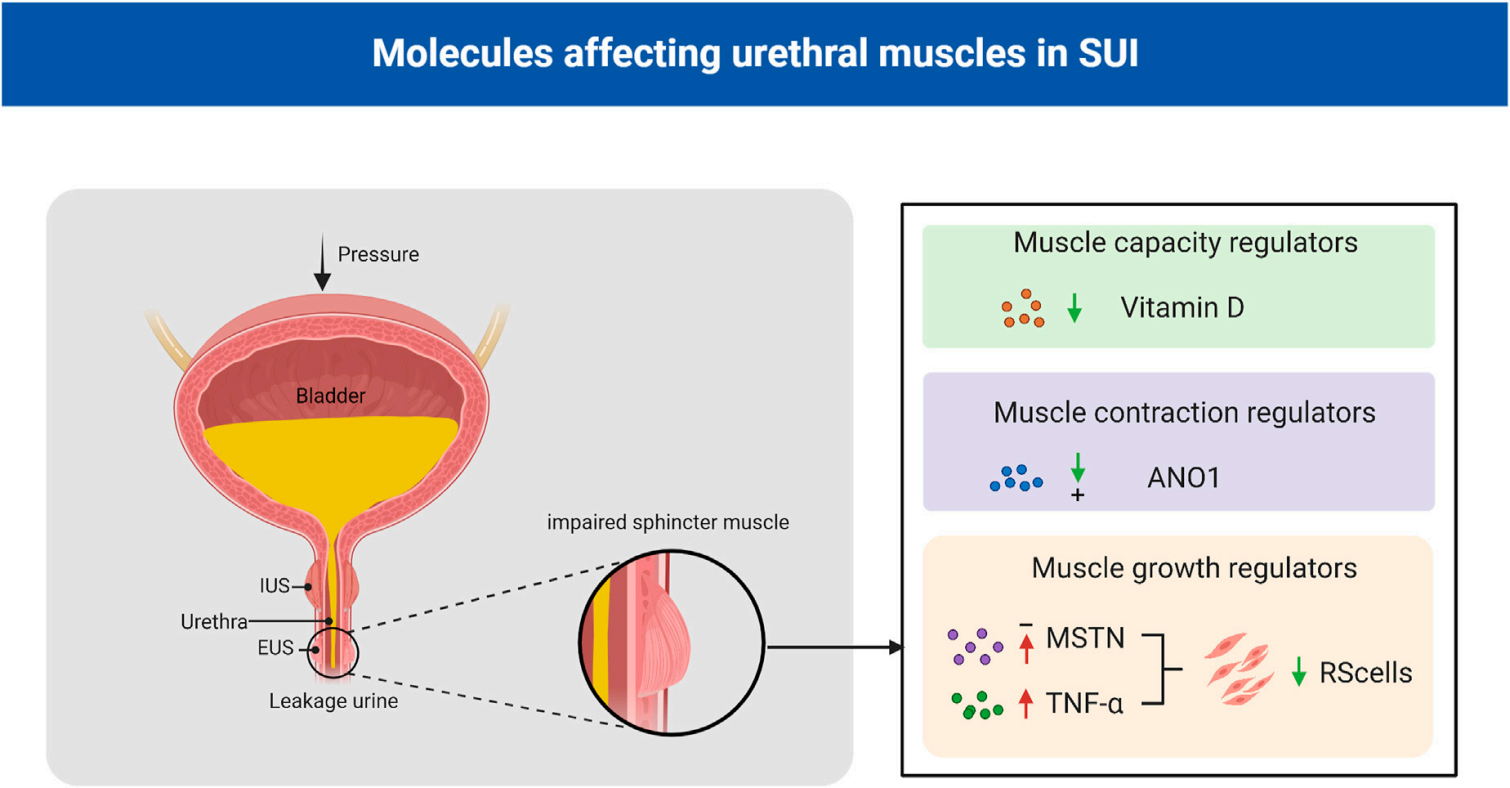
Molecules affecting urethral muscles in SUI. Vitamin D plays an important role in regulating muscle strength and function, and low serum vitamin D levels are associated with an increased risk of SUI; ANO1 is essential for maintaining the spontaneous tone of urethral smooth muscle; MSTN negatively regulated human urethral RS satellite cells proliferation and differentiation and TNF-α decreased the number of human urethral RS cells. EUS: external urethral sphincter; IUS: internal urethral sphincter; MSTN: myostatin; TNF-α: tumor necrosis factor-α; ANO1:anoctamin-1; SUI:stress urinary incontinence; RS: rhabdosphincter.

The SUI level depends on the urethra’s ability of the urethra to maintain a strong urethral closure pressure during intra-abdominal pressure fluctuations. For females, the urethra is short and straight, and the urethral closure pressure could be impaired due to factors such as childbirth. However, the male urethra is divided into three parts, which are long and curved, and the size of the prostate gland increases with age, making males more prone to dysuria rather than incontinence. In male patients, SUI usually results from neurovascular bundle injury during radical prostatectomy or external urethral sphincter injury during transurethral resection or enucleation of the prostate (Koch and Kaufman, 2022). The concerted work of the urethral striated muscle, the urethral smooth muscles, and the vascular elements within the submucosa of the urethra ensure urethral sphincter closure and maintain continence. Some moleculars play a crucial role in maintaining urethral muscles growth, contraction and capacity (Figure 2). A damaged sphincteric unit or support system is all potential causes of an insufficient closure mechanism (Mistry et al., 2020).

### 3.1 Muscle growth regulators

Myostatin (MSTN), which is also known as growth differentiation factor 8 (GDF-8), is a member of the TGF-β superfamily. MSTN plays a role in the control and maintenance of skeletal muscle mass (McPherron et al., 1997). Certain studies have suggested that MSTN negatively regulates human urethral rhabdosphincter satellite cell proliferation and differentiation. Activation of its downstream targets, such as Smad2/3, inhibits myogenesis and promots fibrosis (Akita et al., 2013). Downregulation of MSTN by clustered regularly interspaced short palindromic repeats interference/deactivated Cas9 nuclease-null-kruppel associated box (CRISPRi/dCas9-KRAB)-mediated gene silencing in L6 rat myoblasts and in Zucker rats could significantly enhanced myogenesis *in vitro*, and significant increased the leak point pressure, the thickness of the urethral striated muscle layer, the ratio of urethral striated muscle to smooth muscle, and the number of NMJs in the SUI rat model (Yuan et al., 2020).

Tumor necrosis factor-α (TNF-α) induced apoptosis in murine skeletal muscle cells (Stewart et al., 2004), and systemic concentration of TNF-α increase with age (Krabbe et al., 2004). TNF-α might be involved in age-related decreases in the number of human RS cells and be a causative factor of SUI in the elderly population. Previous studies have reported that TNF-α inhibits the proliferation of striated muscle progenitor cells from the human urethral rhabdosphincter (RS), and a TNF-α antagonist inhibited these effects on these cells (Greiwe et al., 2001; Hanada et al., 2010). In addition, TNF-α was shown to inhibit the myogenic differentiation of human RS progenitor cells through the PI3K and p38-mitogen activated protein-kinase (MAPK) pathways in a dose-dependent manner. Based on this, the research group established an immortalized human RS cell line to identify a possible new therapy for SUI through autologous transplantation of muscle or adipose-derived stem cells (Shinohara et al., 2017).

### 3.2 Muscle contraction regulators

Anoctamin-1 (ANO1), which is also known as transmembrane protein 16A (TMEM16A), has been shown to be a Ca^2+^-activated Cl-channel. ANO1 is essential for a variety of physiological functions, including smooth muscle contraction (Ji et al., 2019). Feng et al. showed that the ryanodine receptor (RyR)-ClCa-voltage-dependent calcium channel (VDCC) signaling system plays a crucial role in maintaining the spontaneous tone (STT) of the female urethral smooth muscles (USM) in mice and female urethra, and the STT of the female urethra is a key factor in maintaining the tone and contraction of the female urethra (Feng et al., 2019). Caffeine, which is a specific ryanodine receptor (RyR) agonist, can increase urethral contractility and intracellular Ca^2+^ concentrations in female mice compared with male mice. Furthermore, EACT, an ANO1 activator, can cause higher intracellular Ca^2+^ increases and stronger currents in female mice than in male mice (Chen et al., 2020). In addition, the urinary retention time was increased, and the urine output was decreased in smooth muscle cell-specific TMEM16A-knockout (TMEM16A smKO) mice (Feng et al., 2019).

### 3.3 Muscle capacity regulators

Vitamin D plays an important role in regulating calcium and bone homeostasis; it can affect muscle strength and function, and low serum vitamin D levels are associated with decreased muscle mass, strength, and performance in older adults (Holick, 2003; Ceglia and Harris, 2013). Evidence suggests that vitamin D primarily affects the diameter and number of type II (fast-twitch) muscle fibers, and myopathies are mainly caused by type IIA muscle fiber atrophy. Type II fibers primarily generate energy under anaerobic conditions for rapid and forceful contractions; therefore, during activities with increased intra-abdominal pressure, atrophy of fast type II fibers may hinder effective urethral closure, leading to SUI (Marques et al., 2010). Studies have shown that in non-pregnant or pregnant women, low vitamin D levels are associated with an increased risk of SUI and there is a positive relationship between prenatal vitamin D levels and postpartum pelvic floor muscle strength and endurance (Badalian and Rosenbaum, 2010; Aydogmus et al., 2015; Navaneethan et al., 2015).

## 4 Connective tissue factors

**FIGURE 3:**
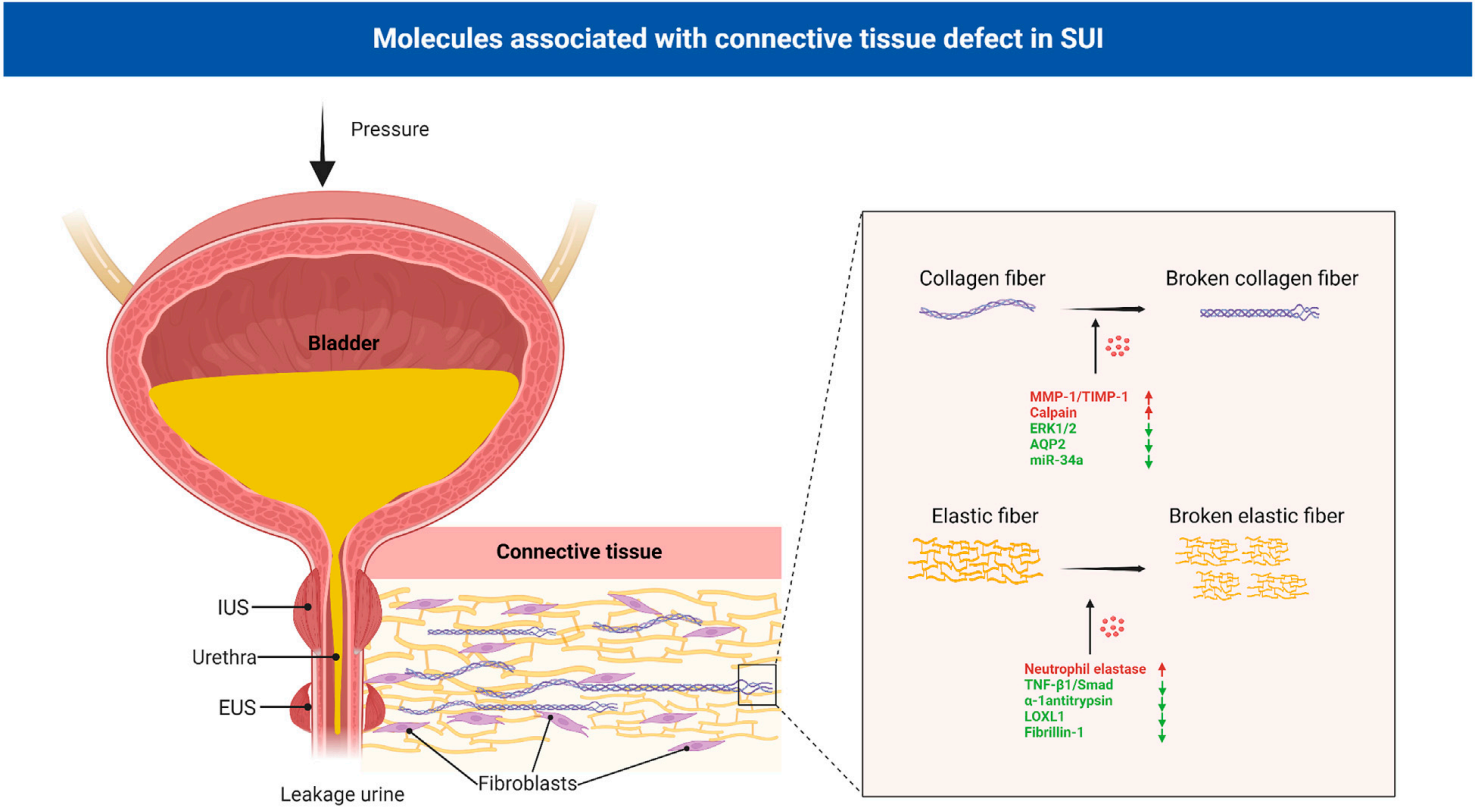
Molecules associated with connective tissue defect in SUI. Connective tissue, mainly contains collagen and elastic fibers, plays an important role in the continence mechanism. In the connective tissue of patients with SUI, collagen and elastic fibers were significantly reduced and damaged. MMP-1, TIMP-1, calpain-2, AQP2, miR-34a and ERK1/2 signaling pathway regulated the metabolism of collagen, and neutrophil elastase, α-1 antitrypsin, LOXL1, fibrillin-1 and TNF-β1/Smad regulated the metabolism of elastic fibers. EUS: external urethral sphincter; IUS: internal urethral sphincter; SUI: stress urinary incontinence; MMPs: matrix metalloproteinases; TIMPs: tissue inhibitors of metalloproteinases; AQPs: aquaporins; LOXL1:lysyl oxidase like-1; TNF-β1:Tumor necrosis factor-β1.

Currently, common theories regarding the pathogenesis of SUI include the integral theory and the Hammock hypothesis, which both stress that structural and functional defects in the supportive tissues of the urethra, including the anterior vaginal wall, contribute to the development of SUI (DeLancey, 1994; Farnsworth, 2002). Effective closure of the female urethra under stress is related to the combined action of various anatomical structures surrounding the urethra, including the connective tissue, pelvic fascia, ligaments and anterior vaginal wall (Delancey and Ashton-Miller, 2004). Connective tissue, which is an essential part, plays an important role in the continence mechanism (Ulmsten and Falconer, 1999). The mechanical stability of the genitourinary tract depends on intact, functional connective tissue to support the bladder neck, urethra and pelvic organs. When connective tissue function is defective, it may lead to the relaxation of the structures involved in the closure of the urethra, making it difficult to close the urethra, and leading to the symptoms of SUI (de Vries et al., 2018). The main constituent in the ligaments and suburethral wall is fibrous connective tissue contains collagen and elastic fibers, proteoglycans and glycoproteins forming a viscoelastic matrix, the extracellular matrix (ECM) (Piculo et al., 2014) (Figure 3). A study showed that there were differences in connective tissue in women with pelvic floor dysfunction, with changes in collagen content and enzymes involved in degradation in pelvic ligament tissue (Chen and Yeh, 2011).

### 4.1 Abnormal collagen metabolism

Collagen forms the matrix of connective tissue and is synthesized by very complex posttranslational processing (Chen et al., 2002). Collagen types I, III and V are the major structural components of the vaginal epithelium and pelvic inner fascia. Type I collagen provides tissue strength, type III collagen helps maintain elasticity and the function of type V collagen is unknown. In the urethral support tissue of patients with SUI, the collagen content was significantly reduced. This finding has important implications in studying the underlying mechanisms of SUI (Song et al., 2007; Sangsawang, 2014). Continuous tissue remodeling makes the relationship between the production and degradation of collagen essential for maintaining tensile strength. Collagen degradation depends on the activity of matrix metalloproteinases (MMPs), a family of structurally related proteins that degrade extracellular matrix and basement membrane components. Collagens are cleaved by the MMP-1, MMP-8 and MMP-13. This process yields two fragments, and these fragments are highly susceptible to MMP-2 and MMP-9 degradation to amino acids. In turn, the activity of MMPs is regulated by the tissue inhibitors of metalloproteinases (TIMPs) (Chen and Yeh, 2011). The balance between MMPs and TIMPs determines collagen breakdown. Women with SUI had an increased ratio of MMP-1 to TIMP-1 mRNA expression and MMP-1 protein expression, but decreased TIMP-1 protein expression in vaginal wall tissue compared with controls (Chen et al., 2002).

Calpain-2 is a neutral protease commonly found in human tissues that is activated by elevated intracellular Ca^2+^ concentrations. It is associated with the degradation of various cytoskeletal proteins and plays an important role in the degradation of myofibers, elastic fibers, and collagen (Li et al., 2017). Compared with those in the controls, the mRNA and protein expression levels of calpain-2 were increased in the urethral tissue of SUI patients (Wu et al., 2010), suggesting that calpain-2 may play a role in the pathogenesis of SUI. Further studies showed that the expression of MMP-1 and collagen I could be regulated by calpain-2 and mediated by miR-93. When miR-93 was overexpressed in SUI, primary fibroblasts restored calpain-2 expression. This finding suggests that calpain-2 is negatively correlated with collagen expression and indicates that miR-93 may mediate the pathogenic mechanism of SUI through calpain-2 (Yang et al., 2018). In addition, a study on the mechanism of electrical stimulation in the treatment of SUI showed that the intracellular Ca^2+^ concentration was markedly enhanced by electrical stimulation. This leads to the activation of the calpain 2/talin 1/integrin β1/transforming growth factor (TGF)-β1 axis, which suppresses cell apoptosis and upregulate collagen (Li Y. et al., 2019). Zhang et al. found that aquaporins (AQPs) are involved in the pathogenesis of female SUI through collagen metabolism during ECM remodeling. It is family of transmembrane proteins, and mediators of transcellular water flow and play important roles in maintaining intra/extracellular fluid homeostasis. The expression of AQP2 in the anterior vaginal wall of women without SUI was significantly increased compared with that of women with SUI. Downregulating AQP2 significantly decreased the mRNA and protein expression of collagen I/III, and overexpressing AQP2 significantly increased the mRNA and protein expression of collagen I/III in fibroblasts (Zhang et al., 2017). A recent study illustrated poor miR-34a expression in SUI patients, miR-34a upregulation inhibited Nampt transcription to accelerate autophagy to suppress ECM degradation (increased collagen and TIMP-1; decreased MMP-2 and MMP-9), thus attenuating SUI (Zhou et al., 2022).

As one of the important MAPK pathways, the extracellular signal-related kinases 1 and 2 (ERK1/2) signaling pathway has been reported to regulate the growth of fibroblasts and collagen at multiple sites (Bhogal and Bona, 2008; Jeon et al., 2009; Choe et al., 2010). Studies have shown that compared with that in the control group, the level of p-ERK1/2 was decreased in female SUI; inhibiting the ERK1/2 signaling pathway can inhibit the synthesis of type I and type III collagen in vaginal wall fibroblasts (Huang et al., 2013). Recent studies also found that the inhibition of the TGF-β1/Smad3 and Nuclear factor-erythroid 2 p45-related factor 2(Nrf2)/antioxidant response element (ARE) signaling pathways also induced a metabolic disorder in the ECM, contributing to the pathogenesis of SUI (Li Y. et al., 2022; Liu et al., 2022).

### 4.2 Abnormal metabolism of elastic fibers

Elastin is primarily formed during fetal development and is rarely synthesized in adult tissues. It provides supporting tissues with the ability to recoil after physical stress and distension, adding the component of resilience to pelvic tissues (Chen et al., 2005). If elastin is damaged or destroyed in adults, it can cause the tissue in which elastin is located to function abnormally. Transmission electron microscopy has been used to examine differences in elastin in the periurethral connective tissue of women with and without SUI, and elastin is irregularly fragmented in SUI patients. This findings indicate that elastin fiber dysfunction may be an important pathology of SUI (Goepel and Thomssen, 2006).

Elastin can be degraded by serine proteases, cysteine proteases, and MMPs. Of the serine proteases, Norepinephrine (NE) is the best known of the degradative enzyme and it is neutralized by α-1 antitrypsin (Wang et al., 2020). Chen et al. studied gene expression in the periurethral connective tissue of the vagina in human women with SUI and found that genes related to elastin metabolism were upregulated in these women compared with controls (Chen et al., 2006). Plasma elastase activity in women with SUI has been shown to be 3 times higher than activity in continent women. Elastolytic activity is increased in pelvic tissues from women with SUI, through an increase in Norepinephrine (NE) activity and a concurrent decrease in α-1 antitrypsin expression (Chen et al., 2004; Chen et al., 2007). Lee et al. found that lysyl oxidase like-1-knockout (LOXL1-KO) mice could not properly assemble elastic fibers and exhibited SUI symptoms (Lee et al., 2008), further indicating that the complete elastic fiber system plays an important role in SUI. Moreover, by constructing a delivery, vaginal distension and ovariectomy (DVDO) rat model of birth trauma, a study showed that birth trauma may activate the expression of urethral elastin through the TGF-β1-associated transcription factors Smad1 and Smad3/4, and estrogen interferes with this signal transduction, resulting in improper assembly of elastic fibers, and weakens the urethra’s ability to withstand the pressure, thus resulting in leakage urine (Lin et al., 2010; Li et al., 2012).

A study showed that the mRNA expression of fibrillin-1 was significantly lower in women with SUI than in women without SUI (Soderberg et al., 2010). Fibrillin is the main component of microfibrils and is important for elastic fiber assembly. Loss of tissue elasticity may lead to hypermobility of the urethra, which in turn results in SUI. In an SUI rat model of urethral injury, pure population of human smooth muscle progenitor cells derived from human pluripotent stem cells (iPSC-pSMCs) were injected around the urethra. The density of elastic fibers in the urethra of SUI rats was increased, and the shape was improved. iPSC-pSMCs can induce ECM remodeling around the urethra in SUI rats, which also suggests that the abnormal elastic fibers may have the same or related molecular mechanism as the ECM abnormalities in SUI. However, this study did not observe improvements in the behavioral marks of SUI rats (Li et al., 2016).

In addition, the macromolecular chondroitin sulfate proteoglycan Vcan and its binding partner hyaluronic acid (HA) accumulate in SUI tissues during inflammation and injury, interfering with elastic fiber assembly in various cases (Keire et al., 2016). A matrix rich in HA and Vcan can promote the expression of chemokines and enhance the binding and retention of inflammatory cells in injured tissues. This process is essential for myofibroblasts (Hattori et al., 2011) and is involved in the development of tissue fibrosis and the occurrence of SUI (Coleman, 2002).

## 5 Role of hormones in SUI

The hormonal status in SUI patients might play an important and potentially modifiable role in preserving and restoring urinary continence (Kreydin et al., 2022). Pelvic floor tissue is regulated by the estrogen and androgen signaling pathways. Recently, the role of androgens in SUI has been explored and shown some preliminary promise. The levator ani muscle and pubocervical fascia are androgen sensitive and contain a large number of androgen receptors. Androgens can promote hypertrophy and hyperplasia in pelvic floor muscles and connective tissue. They cause an increase in fat-free muscle mass, muscle diameter and maximal muscle strength (Fitzgerald, 1994; Castelan et al., 2020). A previous study showed that testosterone levels were lower and SUI complaints were more prevalent in postmenopausal women than in perimenopausal women (Kwon et al., 2014). Kim et al. also reported that low serum testosterone is associated with an increased likelihood of SUI in women (Kim and Kreydin, 2018). Furthermore, a significant association was found between Δ4-androstenedione and the presence of SUI was found. More specifically, women with higher Δ4-androstenedione had lower odds for having SUI (Augoulea et al., 2017). As women age, androgen exposure decreases, which may be another pathophysiological factor leading to the development of SUI. Current evidence supports the ability of androgens to increase pelvic floor muscle mass and strength. Mammadov et al. utilized a rat model to demonstrated that testosterone has both preventive and curative effects on SUI (Mammadov et al., 2011). Some researchers have reported the effect of a selective androgen receptor modulators (SARMs) in the treatment of SUI by enhancing smooth- and striated muscle-mediated urethral function under stress conditions such as sneezing in a rat model (Kadekawa et al., 2020). However, in women, serum androgen levels are low. The effect size related to the serum testosterone difference between women with and without SUI is small, and the effect of Δ4-androstenedione on sex hormone-binding globulin (SHBG) is far less significant than that of endogenous estradiol. In addition, the long-term side effects of androgens and the role of SARMs remain unknown and deserve further study.

The interplay between estrogens and SUI has long been studied with equivocal results. Estrogen has an important impact on the function of the lower genitourinary system and continence mechanism. It raises the sensory threshold of the bladder, increases urethral closure pressure and improves pressure transmission to the proximal urethra (Ahn et al., 2011). The incidence of SUI increases with aging and appears to be associated with age-related declines in estrogen. Some studies have reported decreased E2 levels in postmenopausal and premenopausal women with SUI (Bodner-Adler et al., 2017). In addition, in premenopausal women, downregulation of estrogen receptor α (ERα) expression also plays an important role in SUI. An animal study using an Erα-deficient mouse model, showed a significantly decreased leak point pressure (LPP) in the urodynamics of knockout mice when compared to controls, suggesting an association between ERα and SUI (Ozbek et al., 2014).

However, a deleterious effect on SUI has been observed in some clinical studies. The large SUI subset of the Women’s Health Initiative (WHI) study found that SUI worsened with both estrogen alone and continuous combined estrogen/progestin treatment (Hendrix et al., 2005). This could be caused by several factors. First, oral estrogen therapy has a pronounced enterohepatic first pass effect. The estrogen load on the liver induces an increased production of SHBG. SHBG which binds testosterone and thereby reduces the amount of the free, biologically active hormone, thus worsening SUI through androgen deprivation (Siddle and Versi, 2022). Second, estrogen decreases the total collagen concentration and collagen cross-linking, and increases collagen turnover in peri-urethral tissues (Jackson et al., 2002), thereby affecting urethral motility and urethral closure mechanisms.

## 6 Advances in SUI treatment research

Stem cell therapy has emerged as a promising approach in the field of regenerative medicine in the past few years due to its ability to self-renew, form clonal populations and differentiate into different cell types (Gallo et al., 2019). These cells can restore and maintain normal function by differentiation to replace injured or diseased tissues such as smooth or striated muscle for urethral sphincter regeneration. In addition, stem cells can also exert therapeutic effects by secreting a variety of bioactively angiogenic and cytoprotective factors (Dissaranan et al., 2014). Current research indicates that SUI treatment mainly induces or promotes the formation of normal muscle fibers by various means, thereby replenishing damaged or aging muscle cells to improve muscle function.

### 6.1 Stem cells therapy

**FIGURE 4:**
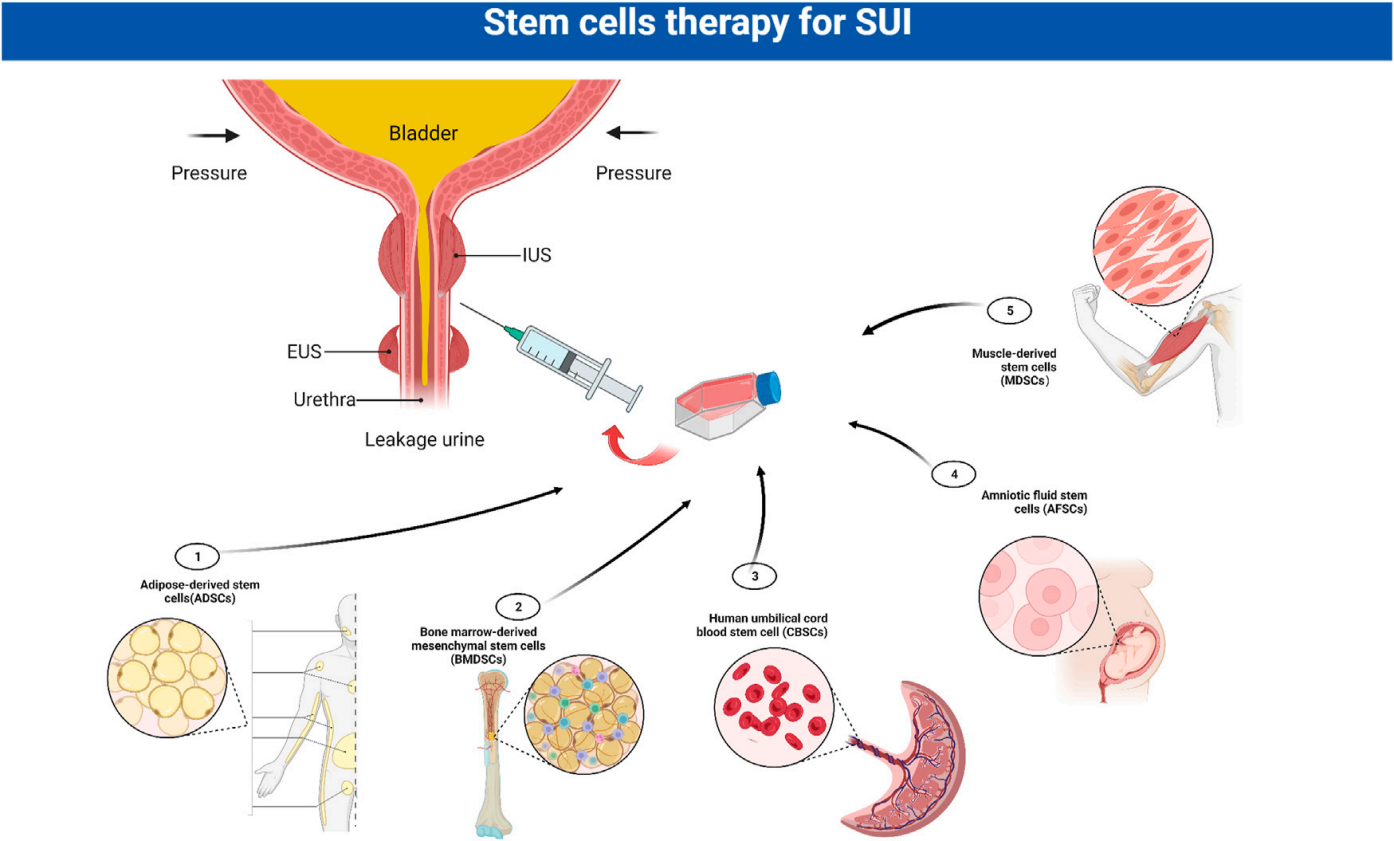
Stem cells in the treatment of SUI. The most widely used stem cells for stress urinary incontinence (SUI) are muscle-derived stem cells (MDSCs), adipose-derived stem cells (ADSCs), bone marrow hematopoietic stem cells (BMDSCs), amniotic fluid stem cells (AFSCs) and cord blood stem cells (CBSCs). After verifying the plasticity of stem cells, they are expanded to a certain number and injected into the urinary system. EUS: external urethral sphincter; IUS: internal urethral sphincter; SUI:stress urinary incontinence.

Stem cells have shown great potential in SUI therapy and have been used in some clinical treatment studies (Table 1). Currently, the most widely used cells are muscle-derived stem cells (MDSCs) and non-muscle-derived stem cells, such as adipose-derived stem cells (ADSCs), bone marrow hematopoietic stem cells (BMDSCs), amniotic fluid stem cells (AFSCs) or cord blood stem cells (CBSCs) (Zhou et al., 2016). After verifying the plasticity of stem cells in proliferating and differentiating into functional cells *in vitro*, they were expanded to a certain number and injected directly, or differentiated into muscle precursor cells *in vitro* and injected, or mixed with some other cells or growth factors and injected into the urinary system or specific surrounding areas (Gallo et al., 2019) (Figure 4). Myogenic differentiation in the damaged EUS can improve or repair damaged striated muscle tone in patients with SUI, thereby improving the function of damaged tissues.

**TABLE 1:** Clinical studies that investigate stem cells therapy for SUI.

Cells type	Authors/Years	Methods of injection	Quantity of cells	Assessment time points	Functional results	Safety results
Autologous muscle derived cells	Carr LK, et al.,/2008	Transurethral injection	18–22×10^6^ cells	1, 3, 6, and 12 months	Improvement in SUI was seen in five of eight women, with one achieving total continence	No serious adverse events were reported
	Sèbe P, et al.,/2011	Intrasphincteric injection	1×10^7^, 2.5×10^7^ and 5×10^7^ cells	1, 2, 3, 6, and 12 months	Quality of life was improved in half of patients but no variation was diagnosed on Qmax measurements	No serious adverse events were reported
	Cornu JN, et al.,/2011	Intrasphincteric injection	1×10^7^, 2.5×10^7^ and 5×10^7^ cells	1, 2, 3, 6, and 12 months	4/12 patients describing reduced urine leakage episodes, 1/12 patient presenting increased maximal closure pressure, and 8/12 patients showing improvement on pad-test	Three cases of urinary tract infection treated by antibiotics
	Carr LK, et al.,/2013	Intrasphincter injection	Low doses: 1, 2, 4, 8 or 16 × 10^6^; high doses: 32, 64 or 128 × 10^6^	1, 3, 6 and 12 months	Greater reduction in pad weight (88.9% vs. 61.5%); greater reduction in diary reported stress leaks (77.8% vs. 53.3%)	Pain and/or bruising at the biopsy site (7.9%) and pain at the injection site (10.5%)
	Peters KM, et al.,/2014	Intrasphincter injection	10, 50, 100 and 200×10^6^ cells	1, 3, 6 and 12 months	All dose groups had significantly fewer diary reported stress leaks at 12 months, high dose group had a statistically significant reduction in mean pad weight	No serious adverse events were reported
	Stangel-Wojcikiewicz K, et al.,/2014	Urethral rhabdosphincter injection	0.6–25 × 10^6^ cells	8 months and 2 years	The 2-year follow-up revealed a 75% success rate, with some patients achieving complete improvement (50%) and some patients achieving partial improvement (25%) on the Gaudenz Questionnaire	No serious adverse events were reported
	Jankowski RJ, et al.,/2018	Intrasphincteric injection	150×10^6^ cells	1, 3, 6, 12 and 24 months	Primary outcome was composite of ≥50% reduction in stress IEF, 24-h or in-office pad weight tests at 12 months	No serious adverse events were reported
	Sharifiaghdas F, et al.,/2019	Transurethral injection	50×10^6^ cells	1, 3, 6, 9, 12, and 24 months	The mean of Qmax decreased in all patients. Improvements in UDI-6 and IIQ-7 scores were recorded at 3- and 6-month, which lasted up to 24 months	No serious adverse events were reported
	Ismail S, et al.,/2020	Intrasphincter injection	150 × 10^6^ ± 20% cells	12 months	Significant increases of sphincter volumes as well as reduction of stress IEF occurred among the AMDC and placebo injection groups with no between-group differences at 12 months	Not given
	Blaganje M, et al.,/2022	Transurethral injection	0.2 × 10^6^ cells	1 week,3.6 months,1,2-year	IEF score, short pad test, quality of life, patient’s and clinician’s perception significantly improved and remained improved after 2 years	Not given
	Strasser H, et al.,/2004	Urethral submucosa and rhabdosphincter injection	Not given	Not given	MUCP, the thickness of postoperative urethral wall and striated sphincter are increased	No serious adverse events were reported
	Mitterberger M, et al.,/2007	Transurethral injection	Fibroblasts: 3.8×10^7^ myoblasts: 2.8×10^7^	1 year	The Incontinence Score and IQOL score was significantly improved. The mean MUCP and the thickness and contractility of the rhabdosphincter all improved. The EMG activity at rest is increased	No serious adverse events were reported
	Blaganje M, et al.,/2012	Intrasphincter injection	1 × 10^6^–5 × 10^7^ cells	6 weeks	The results to the stress test were negative for 78.4% of the patients, 13.5% considered their SUI cured, and 78.4% reported improvement	No serious adverse events were reported
Autologous adipose derived cells	Yamamoto T, et al.,/2010	Transurethral injection	1 ml ADSC fraction and another 4 ml of the fraction was mixed with intact autologous adipose	12 weeks	Urinary incontinence progressively improved. MUCP and functional profile length increased	No serious adverse events were reported
	kuismanen K, et al.,/2014	Transurethral injection	2.5–8.5 × 10^6^ cells	3, 6, and 12 months	After 6 months, 1 of 5 patients displayed a negative cough test with full bladder. At 1 year, the cough test was negative with three patients; two of them were satisfied with the treatment and did not wish further treatment for SUI. Validated questionnaires showed some subjective improvement in all five patients	One patient displayed mild pollacis and dysuria that resolved spontaneously within a week
	Choi JY, et al.,/2016	Transurethral injection	1 mL ADSC fraction and another 4 mL of the fraction was mixed with intact autologous adipose	2, 4, 8, and 12 weeks	Urine leakage volume, subjective symptoms and quality of life were improved. The mean MUCP increased at 12 weeks after injection. MRI showed an increase in functional urethral length	No serious adverse events were reported
	Gotoh M, et al.,/2017	Urethral submucosa and rhabdosphincter injection	1 × 10^7^ cells	52 weeks	37.2% patients displayed improvement in leakage volume at 52 weeks. In the King’s Health Questionnaire, improvement of the quality of life’ scores showed greater improvement in responders, as compared with non-responders	No serious adverse events during the trial
	Arjmand B, et al.,/2017	Transurethral and transvaginal approach	1.18 × 10^7^ cells	2, 6 and 24 weeks	Urinary incontinence significantly decreased through the first two, 6 and 24 weeks after the injection therapy. The difference was significant in pad test results and ICIQ-SF scores; Qmax showed improvement after the study period	One patient displayed slight voiding difficulty, which needed catheterization
Autologous total nucleated cells	Shirvan MK, et al.,/2013	External urethral sphincteric and submucosal injection	TNCs: 6.76 ± 1.22×10^8^ MNCs:1.88 ± 0.32×10^8^; Platelets:3.01 ± 0.11×10^10^	1, 3, and 6 months	ICIQ-UI base line: patients (18.33 ± 0.6), 1 month (1.11 ± 0.5), 3rd and 6th months under one (0.44 ± 0.4); ICIQ-QOL base line: patients (28.8 ± 3.7), 1 month (94.11 ± 12.8), 3rd (96.7 ± 11.8) and 6th months (97.6 ± 13)	None of the patients had voiding dysfunction, urinary retention or urinary tract infection after injection
Autologous mesenchymal stem cells	Garcia-Arranz M, et al.,/2020	Intraurethral injection	Male patient: 20 × 10^6^ or 40 × 10^6^ cells; female patient: 40 × 10^6^ cells	4, 12, 24 weeks, and 1 year	37.5% men and 50% women showed an objective improvement of >50% and a subjective improvement of 70%–80% from baseline	No adverse effects were observed

IEF: incontinence episode frequency; AMDCs: autologous muscle-derived cells; ICIQ-UI:International Consultation on Incontinence Questionnaire-Urinary incontinence; ICIQ-QOL:International Consultation on Incontinence Modular Questionnaire-Quality of Life; IIQ-7:In-continence Impact Questionnaire-7; UDI-6:Urogenital Distress Inventory-6; ICIQ-SF: international consultation on incontinence questionnaire short form; I-QOL: incontinence and quality of life instrument; Qmax: maximum urinary flow rate; MUCP: maximum urethral closing pressure.

#### 6.1.1 MDSCs

MDSCs are currently the most widely used stem cells for the treatment of SUI. These cells are derived from satellite cells which between the basal lamina and sarcolemma of muscle fibers (Morgan and Partridge, 2003). MDSCs can be easily obtained from muscle tissue under local anesthesia. MDSCs are able to differentiate not only into muscle cells, but also to differentiate into neural and endothelial cell lines, making them an ideal material for the treatment of SUI.

The use of injectable cultured MDSCs for the treatment of SUI has been investigated in several animal studies, including those using rodent, porcine, canine and primate models, found good tissue regeneration, improved valsalva leak point pressure (VLPP) and recovered contractile urethral sphincter function (Hillary et al., 2020). The most commonly used animal model is rodents. It was initially reported that sphincter function was improved by injecting MDSCs into pudendal denervated rats (Lee et al., 2004). An early study demonstrated that muscle precursor cells harvested from limb skeletal muscles in mice, may accelerate sphincter muscle repair, as shown by a higher myofiber diameter and number at 7 days. One month after injection, muscle precursor cells were still detectable in the regenerating sphincters and participated in the formation of new myofibers (Yiou et al., 2002). Badra et al. reported that the injections of five million autologous green fluorescent protein labeled skeletal muscle precursor cells resulted in long-term structural and functional restoration of the injured sphincter complex in a monkey model (Badra et al., 2013).

Studies on using MDSCs to treat SUI have reached the clinical level. Several studies have investigated the safety and efficacy of autologous MDSCs therapy among SUI patients (Table 1). A study conducted a 1-year follow-up of eight female SUI patients (42–65 years old) who received MDSCs injection under local anesthesia. This study found that symptoms improved in five (62.5%) SUI patients, and one woman had complete remission (Carr et al., 2008). In the largest series of patients, 123 women with SUI were treated with transurethral ultrasonography-guided injections with autologous myoblasts and fibroblasts obtained from skeletal muscle biopsies. At 1 year after injecting these cells, 79% of women were subjectively completely continent, with significant improvements in the incontinence and quality of life instrument (I-QOL) scores, and the thickness, contractility and electromyographic activity of the rhabdosphincter (Mitterberger et al., 2007). Another large study investigating the use of MDSCs injection for the treatment of SUI, including 80 female patients, was published in 2014. The investigators pooled the data from two trials of autologous MDSCs injections at a dose range of 10 × 10^6^–200 × 10^6^. All treatment groups described subjective improvements sustained over the study period, and higher dose groups had greater percentages of patients with at least a 50% reduction in stress leaks and pad weight (Peters et al., 2014). Recently, a paper summarized 11 studies on the application of MDSCs in the clinical treatment of SUI and found that the median postoperative improvement rate of SUI patients after the injection of MDSCs was 77% (Barakat et al., 2020). Intraurethral injection of MDSCs is a simple surgical procedure that appears safe and effective in women with SUI. However, MDSCs have relatively poor proliferative potential and often require repeated injections to provide sufficient cells. Additionally, treatment efficacy declines significantly over time. Furthermore, the biopsy procedure to obtain a sufficient number of MDSCs is painful, and the cell harvesting procedure, if performed incorrectly, may increase the risk of infection in the patients.

#### 6.1.2 ADSCs

ADSCs originate from the mesoderm and can be repeatedly and abundantly acquired from adipose tissues. ADSCs can proliferate rapidly and robustly *in vitro*, and differentiate into various mesodermal and ectodermal cells, including muscle cells, vascular smooth muscle cells, endothelial cells and neural cells. This is highly relevant for tissue regeneration and revascularization, in the context of a muscular and vascular structure that is denervated and weakened (Roche et al., 2010).

Some studies have used 5-aza-2′-deoxycytidine to induce the differentiation of ADSCs into myoblasts *in vitro*. Subsequently, the induced ADSCs were injected into the posterior muscularis urethra of an SUI rat model, and follow-up analysis demonstrated significant improvements in SUI symptoms (Fu et al., 2010). The implantation of autologous ADSCs into cryo-injured rabbit urethras could promote myogenic differentiation, nerve regeneration and neovascularization in the surrounding tissues and restore urethral function (Silwal Gautam et al., 2014). Furthermore, researchers in one study compared the effects of periurethral and intravenous injection of ADSCs on voiding function and tissue recovery in a SUI rat model. This study found that both two methods of ADSCs injection induced different degrees of recovery of the urethral sphincter, cytokine secretion levels and cell retention rates in the urethral tissues in SUI rats, however, there was no significant difference between the 2 methods (Li G. et al., 2022).

ADSCs have also been utilized in clinical treatment studies for SUI and show great therapeutic potential with a median postoperative improvement rate of 88% after injection (Barakat et al., 2020). One study investigated transurethral ADSC injections in women with SUI. Cells were suspended in a contigen matrix for injection. At 1 year, three of five patients had a negative cough test with the bladder filled to 500 mL. Validated questionnaires showed some subjective improvement in all five patients, but differences in postoperative urodynamic parameters were not significant (Kuismanen et al., 2014). Another study included 45 male SUI patients with mild-to-moderate urine leakage persisting more than 1 year after prostatectomy. Patients demonstrated significant improvements in leakage volume and King’s Health Questionnaire at 52 weeks after ADSCs injection (Gotoh et al., 2020). However, there are still few studies on the clinical application of ADSCs in the treatment of SUI, and there is insufficient evidence to support the long-term efficacy and possible adverse reactions of ADSCs in the treatment of SUI.

#### 6.1.3 Bone marrow-derived stem cells

Bone marrow-derived stem cells (BMDSCs) have promising regenerative properties in preclinical studies. In one preclinical study, BMDSCs were injected into the urethral sphincter of New Zealand White rabbits that were cryo-injured by spraying liquid nitrogen. The proportions of myoglobin- and smooth muscle actin-expressing areas in cell-implanted regions were significantly higher than those of in the controls. By 14 days, these differentiated cells formed contacts with similar cells, creating layered muscle structures. At that time, the leak point pressure of the cell-implanted rabbits was significantly higher than that of the controls (Imamura et al., 2011). In another study, BMDSCs transplantation enhanced closing pressure and leak point pressure in a female urinary incontinence Sprague-Dawley rat model (Kim et al., 2011). However, bone marrow procurement requires general or spinal anesthesia, and yields a low number of stem cells upon processing. No trial in humans on the effect of BMDSCs injection for the treatment of SUI has been reported to date.

#### 6.1.4 CBSCs and AFSCs

Although MDSCs and ADSCs can be obtained in large quantities under local anesthesia, collection remains an invasive procedure with the risk of morbidity. Stem cells can also be obtained from human cord blood or amniotic fluid. These cells have a high proliferation rate, induce immune tolerance, display embryonic stem cell properties and are able to differentiate into cells representing all three embryonic germ layers including cells of adipogenic, osteogenic, myogenic, endothelial, neural, and hepatic lineages (De Coppi et al., 2007). Peri-urethral injections of CBSCs for the treatment of SUI were investigated in one study, which included 39 patients with SUI. 29 patients (83%) were more than 50% satisfied according to the patient’s satisfaction results after 3 months, and 26 (72.2%) continuously showed more than 50% improvement after 12 months. Intrinsic sphincter deficiency and mixed stress incontinency improved in the ten patients evaluated by urodynamic study.

AFSCs displayed MSC characteristics and could differentiate into cells of the myogenic lineage. Kim et al. evaluated the therapeutic feasibility of periurethral injection of AFSCs in an SUI animal model. Four weeks after injection, the mean leak point pressure and closing pressure were significantly increased in the AFSC-injected group compared with the control group. Nerve regeneration and neuromuscular junction formation of injected AFSCs *in vivo* were confirmed by the expression of neuronal markers and acetylcholine receptors (Kim et al., 2012).

In addition, investigators continue to identify novel, less-invasive cell sources for SUI, including cells derived from hair follicles, menstrual fluid, and urine (Gallo et al., 2019). However, as there have been few studies as of July 2022, there are currently fewer than 20 clinical trials registered internationally (Table 2). Additional studies are essential to further investigate the efficacy and safety of stem cells in the treatment of SUI. Cell regeneration therapy in the treatment of SUI is currently progressing well. However, the survival time after stem cell transplantation, the side effects such as tissue swelling, and the optimal amounts of cells for injection still require more comprehensive and careful evaluation, and an optimization of technical processes is needed.

**TABLE 2:** Clinical progress of cell therapy in stress urinary incontinence patients.

NCT number	Cell therapy	Phase	Study start date	Study completion date	Status	Outcome
NCT00847535	AMDCs	Phase 2	9 October 2008	2 November 2011	Complete	No adverse events reported during the study were adjudicated as AMDCs product-related
NCT01008943	AMDCs	Phase 2	2 June 2010	21 September 2012	Complete	No adverse events reported during the study were adjudicated as AMDCs product-related
NCT01799694	Autologous adipose-derived stem cells	Phase 2	July 2011	February 2013	Complete	No Results Posted
NCT01648491	Autologous stem cells	Not Applicable	October 2011	January 2016	Complete	No occurrence of adverse events, and the quality of life’ score described by the patient’s response on the Global Response Assessment was improved
NCT01382602	AMDCs	Phase 3	4 November 2011	29 January 2017	Complete	8/93 (8.60%) suffered Nausea in AMDC-USR compared with 0/50 (0.00%) in Placebo group; 5/93 (5.38%) suffered injection site pain in AMDC-USR compared with 1/50 (2.00%) in Placebo group; 12/93 (12.90%) suffered urinary tract infection in AMDC-USR compared with 5/50 (10.00%) in Placebo group; 5/93 (5.38%) suffered dysuria in AMDC-USR compared with 3/50 (6.00%) in Placebo group; 2/93 (2.15%) suffered pollakiuria in AMDC-USR compared with 4/50 (8.00%) in Placebo group
NCT01804153	Adipose-derived stem cells	Phase 1/Phase2	September 2012	April 2014	Unknown status	No Results Posted
NCT01963455	AMDCs	Phase 1	January 2013	September 2014	Completed	No Results Posted
NCT01850342	Fat tissue micrograft mixed with adipose-derived regenerative cells	Phase 1/Phase 2	May 2013	December 2014	Unknown status	No Results Posted
NCT02156934	AMDCs	Phase 2	January 2014	December 2015	Completed	No Results Posted
NCT02291432	AMDCs	Phase 1/Phase2	19 February 2015	28 June 2019	Complete	2/25 suffered product-related adverse events and 12/25 suffered biopsy procedure-related adverse events, no injection procedure-related adverse events were reported. After 3 months therapy, the 24 h pad weight reached the minimum value of 59.5g, 17/23 had≥50% reduction in 24 h pad weight and 15/23 felt better in patient global impression of improvement questionnaire after 12 months treatment
NCT02529865	Autologous adipose-derived regenerative cells	Phase 3	29 July 2015	7 March 2019	Complete	No Results Posted
NCT02334878	Autologous bone marrow-derived mesenchymal stem cells	Phase 3	1 October 2015	1 December 2016	Complete	No Results Posted
NCT04446884	Autologous adipose-derived mesenchymal stem cells	Phase 1	1 March 2018	31 December 2019	Complete	No Results Posted
NCT03104517	AMDCs	Phase 3	23 April 2019	February 2026	Recruiting	Recruiting
NCT04426643	Autologous adipose-derived mesenchymal stem cells	Phase 1/Phase2	1 August 2020	31 March 2021	Complete	No Results Posted
NCT03997318	AMDCs	Phase 3	June 2023	June 2025	Not yet recruiting	Not yet recruiting
NCT04729582	AMDCs	Phase 1/Phase2	May 2023	October 2026	Not yet recruiting	Not yet recruiting

AMDCs: Autologous muscle derived cells; AMDC-USR:autologous muscle derived cells for urinary sphincter repair.

ClinicalTrials.gov database: https://clinicaltrials.gov/

### 6.2 Exosomes

Using exosomes from stem cells to promote cell differentiation is a novel regenerative strategy. Exosomes are membrane-wrapped microcontainers that are separated from cell membranes by exocytosis; they have a diameter of approximately 30–150 nm. Exosomes are able to transport proteins, cytokines and mRNA to target cells and exert effects (Wu et al., 2019). Exosomes secreted by stem cells have been shown to have stem cell-like functions; they effectively prevent tissue damage and repair impaired tissues. Furthermore, they avoid the potential risks of cell transplantation, such as immunogenicity, tumorigenicity, or vascular obstruction. Exosomes containing soluble cytokines can be isolated by the ultrafiltration and centrifugation of cell culture supernatants and the selective separation of samples using ultrafiltration membranes with different relative molecular weight cutoffs (MWCOs) (Phetfong et al., 2022).

The treatment of SUI with exosomes is one of the emerging strategies in recent years. Initial studies showed that ADSC-derived exosomes could enhance the growth of skeletal muscle and Schwann cell lines in a dose-dependent manner. *In vivo* experiments showed that ADSC-derived exosomes could improve urethral function and histology after SUI, and the performance was slightly better than that of ADSCs (Ni et al., 2018). In addition to being able to differentiate into skeletal muscle cells, exosomes can also promote the synthesis of collagen fibers *in vitro*. Studies have reported that exosomes from mesenchymal stem cells could regulate the expression of TIMP-1 and MMP-1, promoting collagen synthesis and inhibiting collagen degradation. Thus, this may be a novel treatment strategy for SUI (Liu et al., 2018). In addition to exosomes secreted by common stem cells (Zhou et al., 2021), Schwann cell-derived exosomes (Hu et al., 2019; Wu et al., 2019; Huang et al., 2021) have also shown potential in the treatment of SUI *in vivo*. However, there have been few studies on the treatment of SUI with exosomes. The curative effect on clinical patients has not been examined, and further research is needed to gain a deeper understanding of exosomes.

### 6.3 Regulation of gene expression

With the increasing understanding of the molecular pathogenesis of SUI, some certain molecules have been confirmed to be involved in the pathophysiological process of the occurrence and development of SUI. Regulating the expression of pathogenic molecules in SUI to restore damaged tissue function is a feasible treatment strategy. In addition to the aforementioned direct silencing of MSTN expression using CRISPR/Cas9 technology to restore urination parameters in SUI rats (Yuan et al., 2020), other transgenic methods have also been applied in the treatment of SUI. The chemokine stromal derived factor-1(SDF-1) can enhance tissue regeneration through stem cells chemotaxis, neovascularization, and neuronal regeneration. The injection of the SDF-1 overexpression plasmid was shown to improve continence function in SUI female rat model. The increased amount of urethral sphincter muscle and higher vascular density provide a potential strategy for the treatment of SUI (Khalifa et al., 2020).

Oxidative stress and inflammatory pathway activation are important mechanisms for the occurrence of SUI, and increased apoptosis and oxidative damage can be found in fibroblasts in a mouse model of SUI induced by mechanical injury (Liu et al., 2022). Nuclear factor erythroid 2-related factor 2 (Nrf2) is a widely used inducer of antioxidant genes that has been shown to reduce oxidative damage and apoptosis. The Nrf2 gene was overexpressed by lentivirus transfection in a mouse model of vaginal dilatation (VD)-induced SUI, and Nrf2 overexpression significantly reduced VD-induced vaginal anterior wall abnormalities. Thus, this may be a promising treatment strategy for mechanical trauma-related SUI (Tang et al., 2019).

Gene regulation is not always used as a monotherapy for SUI, and can also be used in combination with other regimens, such as cell therapy. Lentiviral vector-mediated Smad3 shRNA blockade in SUI rats has been shown to inhibit the differentiation of MDSCs into fibroblasts (but without affecting their myogenic differentiation), which can improve MDSC-mediated repair of urinary sphincter function (Wang et al., 2011). Similarly, Haralampieva D et al. overexpressed human peroxisome proliferator-activated receptor gamma coactivator 1-α (hPGC-1α) in human muscle precursor cells (hMPCs) with an adenovirus. hPGC-1α-overexpressing hMPCs were injected into injured skeletal muscle. hMPCs could significantly promoted muscle regeneration after injury, reduced the expression of proinflammatory cytokines (TNF-α) and enhanced the healing process. (Haralampieva et al., 2018).

## 7 Conclusion

The main pathological mechanisms of SUI are the dysfunction of the nervous system regulating the continence structure, the functional defects in the urethral support structure, and the decreased function of the urethral sphincter, resulting in the inability of the urethra to maintain closure when the abdominal pressure increases. The occurrence of SUI may be affected by a variety of molecules, including neurogenic, muscle-derived and connective tissue-derived molecules. Although the current treatments include conservative and surgical treatment, only 25% of women with SUI currently receive treatment due to the low efficacy of conservative treatment and complications associated with surgery (Lukacz et al., 2017). An increasing number of researchers are beginning to pay attention to new treatment methods. With the continuous advancement of technology, the development of various technologies, including cell therapy, exosome differentiation, gene regulation and other technologies, will provide broad prospects for the treatment of SUI. Most of the research is limited to the cell or animal research stage. Although some studies also involve clinical research, the observation time is short. Additionally, the long-term efficacy and safety of new treatment methods still need further careful and long-term evaluation.
